# Alterations in antioxidant system, mitochondrial biogenesis and autophagy in preeclamptic myometrium

**DOI:** 10.1016/j.bbacli.2017.06.002

**Published:** 2017-07-03

**Authors:** Polina A. Vishnyakova, Maria A. Volodina, Nadezhda V. Tarasova, Maria V. Marey, Natalya E. Kan, Zulfiya S. Khodzhaeva, Mikhail Yu. Vysokikh, Gennady T. Sukhikh

**Affiliations:** a Research Center for Obstetrics, Gynecology and Perinatology, Ministry of Healthcare of the Russian Federation, 4, Oparina street, Moscow 117997, Russia; b Belozerskii Institute of Physico-chemical Biology, Moscow State University, Leninskie gory 1, Moscow 119992, Russia

**Keywords:** Preeclampsia, Myometrium, Oxidative stress, Mitochondria, Autophagy

## Abstract

Preeclampsia is a pregnancy complication which causes significant maternal and fetal morbidity and mortality worldwide. Although intensive research has been performed in the last 40 years, the pathology of preeclampsia is still poorly understood. The present work is a comparative study of the myometrium of women with normal pregnancy, and those with late- and early-onset preeclampsia (n = 10 for each group). We observed significant changes in the levels of antioxidant enzymes, markers of mitochondrial biogenesis and autophagy proteins in preeclamptic myometrium. Levels of superoxide dismutase 1 and catalase were lower in both preeclamptic groups than the control group. In late-onset preeclampsia, expression levels of essential mitochondria-related proteins VDAC1, TFAM, hexokinase 1, PGC-1α and PGC-1β, and autophagy marker LC3A, were significantly elevated. In the myometrium of the early-onset preeclampsia group OPA1 and Bcl-2 were up-regulated compared to those of the control (p < 0.05). These findings suggest that crucial molecular changes in the maternal myometrium occur with the development of preeclampsia.

## Highlights

• Increases in VDAC1, TFAM, HK1, PGC-1α and PGC-1β levels in late-onset preeclamptic myometrium
• Autophagy marker LC3A level was higher in the late-onset preeclampsia group.
• In early-onset preeclamptic myometrium there is an evidence of enhanced mitochondrial fusion.

## 1 Introduction

Preeclampsia (PE) is a multisystem disorder which affects approximately 6% of pregnant women worldwide, and still remains a leading cause of maternal and perinatal morbidity and mortality [1], [2]. Despite the large body of data on the molecular changes associated with PE, its etiology is still poorly understood. There are two clinically-distinct PE phenotypes that vary in the time of onset: early-onset PE (eoPE), which occurs before 34 weeks, and late-onset PE (loPE), which takes place after 34 weeks of gestation [3]. PE is thought to be associated with impaired trophoblast invasion into the myometrial segment of the spiral artery [4], [5], [6]. Subsequent disturbance of placental oxygenation results in permanent ischemia/reperfusion and induction of oxidative stress in the placenta and maternal blood [7], [8], [9], [10]. The present study is an investigation into myometrial tissue from patients with normal pregnancy and those with PE. Myometrium is an uterine muscle, composed of three poorly defined layers (inner, outer and middle), and is rich in blood vessels [11]. A number of studies have demonstrated alterations in the expression of vascular tension modulators in myometrial tissue in PE [12], [13], [14]. However, very few studies have focused on the antioxidant system, mitochondrial apparatus and autophagy in myometrium, despite them being highly interconnected [15]. Free radicals, derived from preeclamptic placenta, induce the circulation of reactive oxygen species (ROS) and oxidation products of biomolecules in the blood. This may affect the functionality of myometrium and endothelial cells by influencing a wide range of cellular processes [10]. Since mitochondria and NADPH oxidase are considered major sources of ROS in PE placentas [16], [17], it is important to evaluate the expression of antioxidant enzymes, state of ROS-sensitive mitochondrial network [18], and level of autophagy in the neighbouring myometrium.

## 2 Material and methods

### 2.1 Ethics statement

All procedures and experimental protocols involving myometrial tissue were conducted in accordance with the Declaration of Helsinki, Guidelines for Good Clinical Practice and Committee on Biomedical Research Ethics of Research Center for Obstetrics, Gynecology and Perinatology, Ministry of Healthcare of the Russian Federation. All the patients signed informed consent in accordance with the Ethics Committee requirements and Helsinki Declaration of the World Medical Association.

### 2.2 Sample collection

Myometrial samples were collected immediately after delivery by elective caesarean section, proposed on clinical grounds for women with normal pregnancies, eoPE or loPE, in the Research Center for Obstetrics, Gynecology and Perinatology, Moscow. PE was diagnosed according to common medical criteria [3]. Myometrial biopsy (0.5 × 0.5 × 0.5 cm) was obtained from the upper edge of lower segment uterine incision, snap frozen in liquid nitrogen and stored at − 80 °C until used.

### 2.3 RNA extraction and reverse transcription

Total RNA was isolated using Extract RNA Reagent (Evrogen, Russia) after homogenisation of myometrial tissue in liquid nitrogen. RNA concentration and 260/280 ratio was measured using a spectrophotometer DS-11 (DeNovix, USA). The integrity of RNA was confirmed by 1.5% agarose gel electrophoresis. For the reverse transcription reaction, 0.5 μg of total RNA was reverse transcribed using the MMLV-RT kit (Evrogen, Russia) with random hexamer primers.

### 2.4 Quantitative real-time PCR

Quantification of mRNA was conducted using DT-96 Real-Time Detection Thermocycler (DNA-Technology LLC, Russia). The reactions were carried out in duplicate in volumes of 10 μl, containing 50 ng of cDNA, 300 nM of each primer, and 2 μl of 5xSybrGreen-mix (Evrogen, Russia). All primers (Supplementary Table S1) were generated by Primer-BLAST [19]. Specificity of the amplified fragments was confirmed by melting curve analysis and electrophoresis of the PCR products on 1.5% agarose gel. The PCR program consisted of an initial step at 95 °C for 5 min, followed by 45 cycles of denaturation at 95 °C for 10 s, annealing at 60 °C for 20 s, and elongation at 67 °C for 20 s, followed by melting at a gradient from 65 °C to 95 °C. Relative gene expression was determined as the ratio of the target gene to the internal reference gene expression (β-actin) based on Ct values, using QGENE software [20].

### 2.5 mtDNA copy number

DNA extraction from myometrial homogenate was conducted using DNA Extran-2 kit (Syntol, Russia). The mtDNA content was measured by Real-Time PCR, normalising the quantity of a non-polymorphic region of D-loop with a single copy nuclear gene (β-2-microglobulin). Primer sequences are shown in Supplementary Table S1. 100 ng of total DNA was analysed in duplicate under the following conditions: 50 °C for 2 min, 95 °C for 20 s, followed by 45 cycles of denaturation at 95 °C for 15 s, annealing at 63 °C for 20 s, and elongation at 67 °C for 20 s, followed by melting at a gradient from 65 °C to 95 °C. Relative quantification values were calculated by the 2^− ΔCt^ method [21].

### 2.6 Activity of citrate synthase

Citrate synthase activity was determined in myometrial tissue homogenate at a wavelength of 412 nm as described by Eigentler et al. [22].

### 2.7 Western blot analysis

Sample preparation and immunoblotting were performed as previously described [23]. Membranes were incubated with primary antibodies (anti-SOD1–ab13498; anti-SOD2–ab16956; anti-catalase- ab76024; anti-GPx1–ab108427; anti-VDAC1–ab154856; anti-TFAM–ab155240; anti-PGC-1α–ab77210; anti-PGC-1β–ab176328; anti-OPA1–ab119685; anti-MFN1–ab57602; anti-MFN2–ab56889; anti-DRP1–ab56788; anti-LC3A–ab52628; anti-HK1–ab55144, all–Abcam, USA; anti-beta-actin–MA5-15739, anti-Bcl-2-13-8800, Invitrogen, USA) overnight at 4 °C with gentle shaking. After washing, the membranes were incubated with peroxidase-conjugated secondary antibodies for 1 h at room temperature. Target proteins were detected using Novex ECL Kit (Invitrogen, USA) in ChemiDoc station (Biorad, USA). Optical densities of the protein bands were measured using ImageLab Software. Protein content was normalised on β-actin.

### 2.8 Statistical analysis

Data is presented as mean ± standard error mean (SEM). The Shapiro-Wilk normality test was used to estimate distribution [24]. One-way analysis of variance (ANOVA) followed by the Tukey's post-hoc test was used to identify differences among multiple groups with normal distribution. One-way Kruskal-Wallis non-parametric ANOVA followed by the post-hoc Dunn test was used to calculate statistical differences for non-normal distributions. All calculations were performed by Prism 7.0 software (GraphPad, USA) and Website VassarStats for Statistical Computation (www.vassarstats.net). p-Value < 0.05 was considered significant and was indicative of the differences in comparison to control.

## 3 Results

### 3.1 Clinical data

Clinical and demographic data of the study patients are summarized in Table 1. Women with early-onset and late-onset PE showed significantly increased systolic and diastolic blood pressure, and proteinuria, in comparison with normal pregnancies. Lower baby weight and intrauterine growth restriction (IUGR) were observed in both PE types.

**Table 1:** Clinical and demographic characteristics of patients. Data is listed as mean ± SEM.

Characteristics	Control	loPE	eoPE
Number	10	10	10
Maternal age, years	32.7 ± 1.4	30.2 ± 1.1	33.7 ± 1.2
Gestational age at delivery, weeks	39.8 ± 0.1	38.0 ± 0.3	31.8 ± 0.4⁎
Body mass index before delivery, kg/m^2^	26.6 ± 1.0	28.7 ± 1.0	28.9 ± 1.5
Systolic blood pressure, mm Hg	112.3 ± 1.2	152.2 ± 3.1⁎	163.7 ± 4.5⁎
Diastolic blood pressure, mm Hg	73.2 ± 1.2	95.7 ± 2.4⁎	99.9 ± 2.1⁎
Proteinuria, mg/dL	ND	100.9 ± 50.0⁎	168.2 ± 40.2⁎
Sex of the baby (male/female), %	70/30	60/40	50/50
Intrauterine growth restriction, %	ND	30⁎	60⁎
Baby mass, g	3395.5 ± 128.1	2799.3 ± 192.8⁎	1523.1 ± 146.9⁎

ND–not detected.

⁎ р < 0.05 versus control.

### 3.2 Decline of antioxidant system in preeclamptic myometrium

**Fig. 1:**
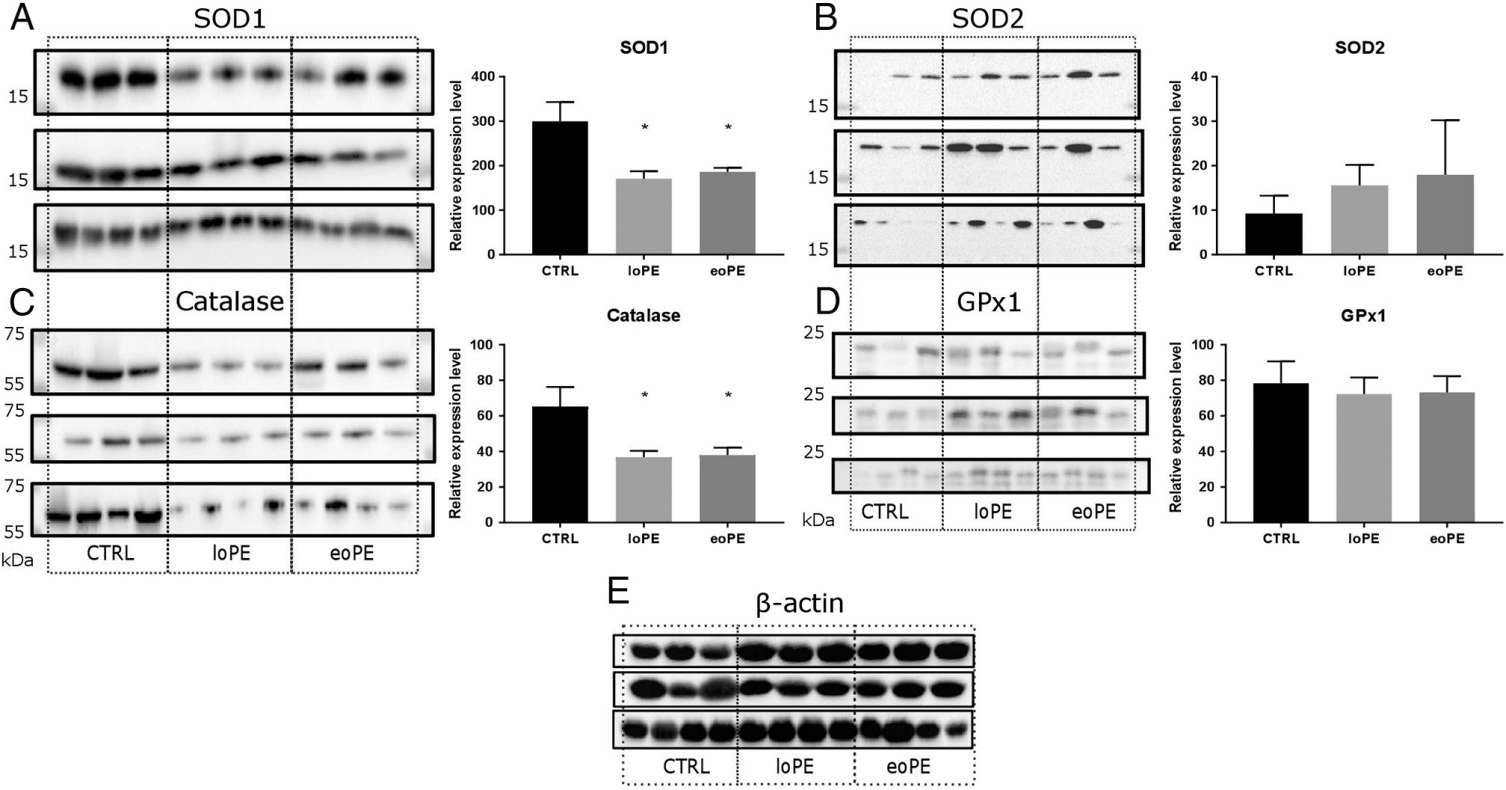
Antioxidant enzymes content in myometrium. Relative level of SOD1 (A), SOD2 (B), catalase (C) and GPx1 (D) in myometrium of control (CTRL) group and preeclamptic pregnancies: late-onset PE – loPE, early-onset PE – eoPE (n = 10 for each group), according to western blot analysis (left side – membrane staining, right side – protein level relative to β-actin). β-actin was used as a loading control (E). *p < 0.05 versus control.

To evaluate the antioxidant system in preeclamptic and control myometrium, we measured protein expression of four essential enzymes that are responsible for maintaining a physiological ROS level: cytoplasmic superoxide dismutase 1 (SOD1), mitochondrial superoxide dismutase 2 (SOD2), catalase and glutathione peroxidase 1 (GPx1). Relative level of SOD1 (Fig. 1A) was significantly decreased in both PE groups compared to the control (1.6-fold for eoPE, p = 0.017; 1.7-fold for loPE, p = 0.006). In contrast, the production of SOD2 in preeclamptic myometrium and normal myometrium was similar (Fig. 1B). The level of catalase (Fig. 1C) was significantly lower in preeclamptic myometrium; we observed a 1.8-fold decrease of catalase expression in both PE groups (p = 0.020 for both). We did not find any significant differences in content of GPx1, another important antioxidant protein (Fig. 1D).

### 3.3 Mitochondrial biogenesis at PE

**Fig. 2:**
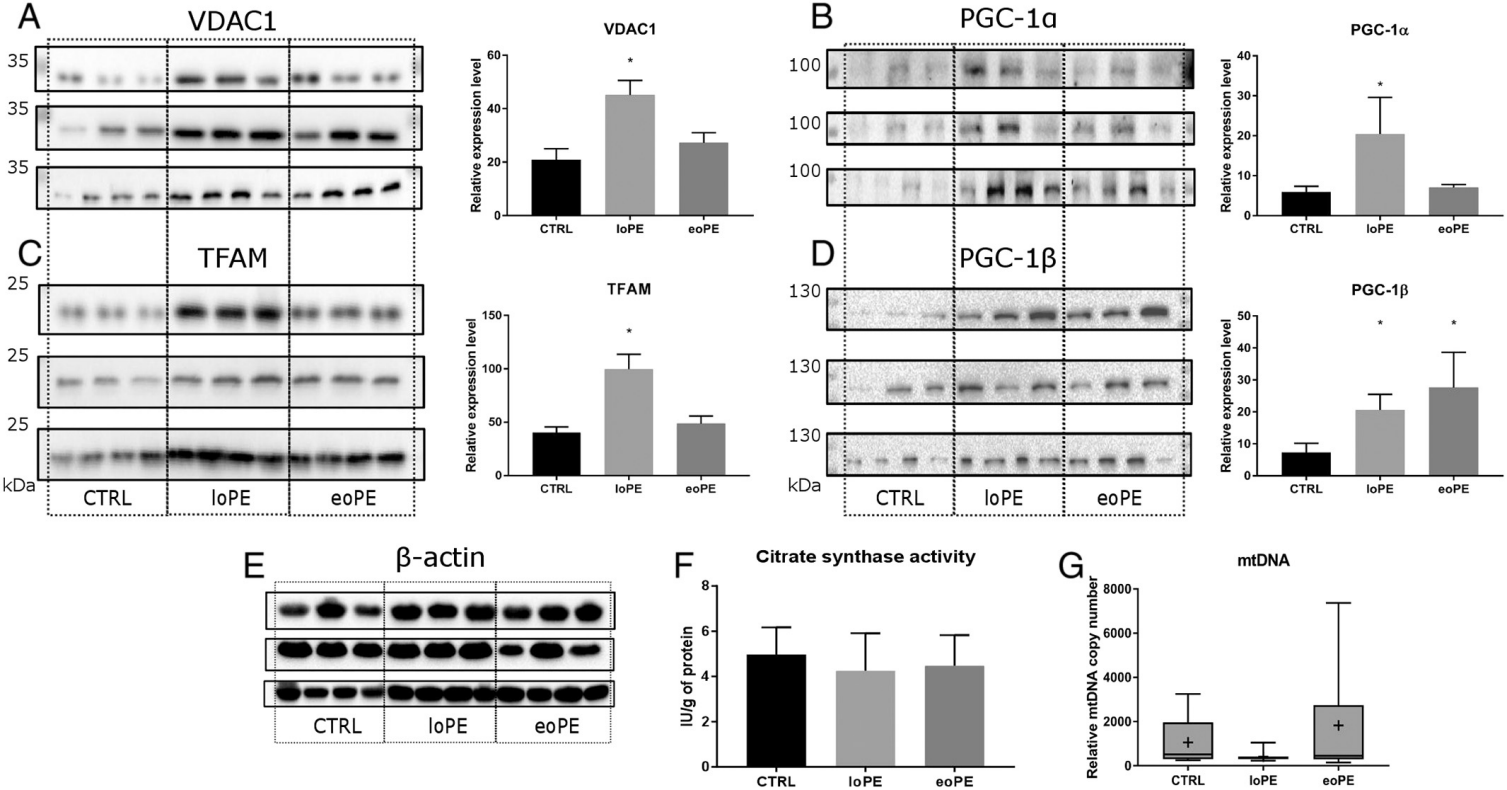
Mitochondrial biogenesis in myometrium. Relative level of VDAC1 (A), PGC-1α (B), TFAM (C) and PGC-1β (D) in myometrium from normal, early- and late-onset PE pregnancies: late-onset PE – loPE, early-onset PE – eoPE (n = 10 for each group), according to western blot analysis (left side – membrane staining, right side – protein level relative to β-actin). β-actin was used as a loading control (E). Results of citrate synthase activity assay (F). Relative mtDNA copy number according to PCR analysis (G). The median (line), mean (cross) and 25–75% interquartile range are shown. *p < 0.05 versus control.

Mitochondria, especially the respiratory chain, remain the major source of cellular ROS. As such, we investigated whether markers of mitochondrial quantity and biogenesis in the myometrium change as a result of PE development. The relative level of VDAC1, an important mitochondrial channel, was two-fold higher in the myometrium of the loPE group (Fig. 2A), than that of the control group (p = 0.002). The level of TFAM, another mitochondrial protein responsible for mtDNA transcription and replication, was also higher in the loPE group (Fig. 2C); a 2.5-fold increase in its expression was observed (p = 0.001). The production of mitochondrial biogenesis inducers PGC-1α and PGC-1β was then assessed (Fig. 2B and D). We found significant increase in the PGC-1α protein level in the loPE group (2.4-fold, p = 0.040), and the PGC-1β in both PE groups (3.8-fold for eoPE (p = 0.014), and 2.8-fold for loPE (p = 0.011)). Interestingly, changes in the gene expression level of NRF1 and NRF2 (Nuclear Respiratory Factors that are downstream targets of PGC-1α and PGC-1β (S1 Fig.)) were not evident. Moreover, no significant differences were observed in the mitochondrial content tests of citrate synthase activity and mtDNA copy number (Fig. 2F and G).

### 3.4 Fusion and fission

**Fig. 3:**
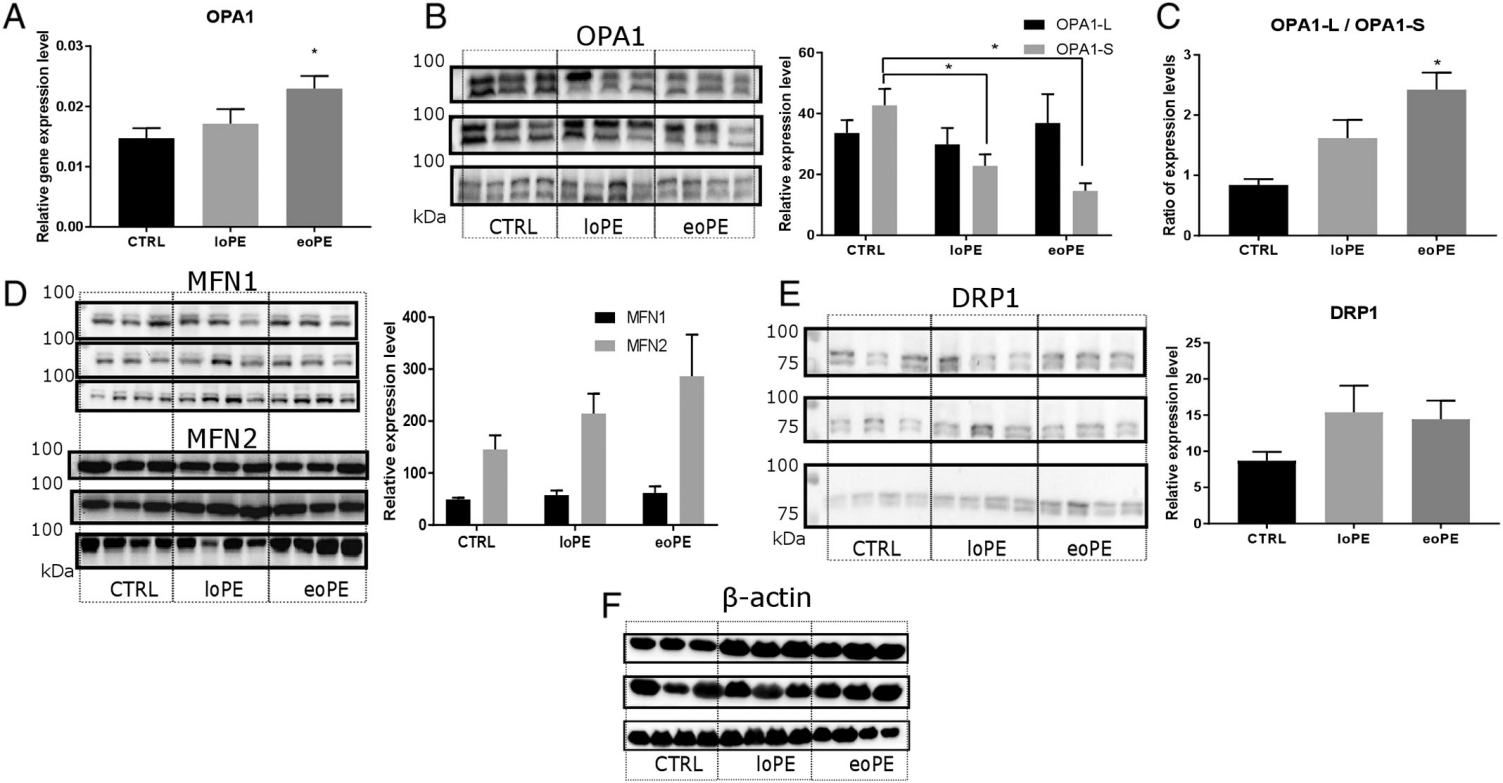
Mitochondrial fusion and fission in myometrium. Relative gene expression level of OPA1, normalised on β-actin (A), according to PCR analysis. Protein level of OPA1 (L- and S-forms: L-upper band, S-lower band) (B), MFN1 and MFN2 (D), DRP1 (E) in myometrium from normal and preeclamptic pregnancies: late-onset PE – loPE, early-onset PE – eoPE (n = 10 for each group), according to western blot analysis (left side – membrane staining, right side – protein level relative to β-actin). Ratio of OPA1-L to OPA1-S levels (C). β-actin was used as a loading control (F). *p < 0.05 versus control.

The processes of mitochondrial elongation and fragmentation are highly sensitive to microenvironment and intracellular changes. We observed significant 1.6-fold increase in the mRNA level of the important profusion agent OPA1 in the eoPE group (p = 0.023), compared to the control (Fig. 3A). With regard to protein content level, significant decreases in the cleaved OPA1 form (S-OPA1) was observed in both PE groups (Fig. 3B). The ratio of the large OPA1 form (L-OPA1) to S-OPA1 reflects the prevalence of fusion to fission, and was 3-fold higher in the eoPE group (p < 0.01) (Fig. 3C). No differences in the expression of MFN1 and MFN2 (proteins involved in the fusion of outer mitochondrial membranes), and DRP1 protein levels (main agent that promotes mitochondrial fission) were evident (Fig. 3D and E).

### 3.5 Autophagy is enhanced in loPE myometrium

**Fig. 4:**
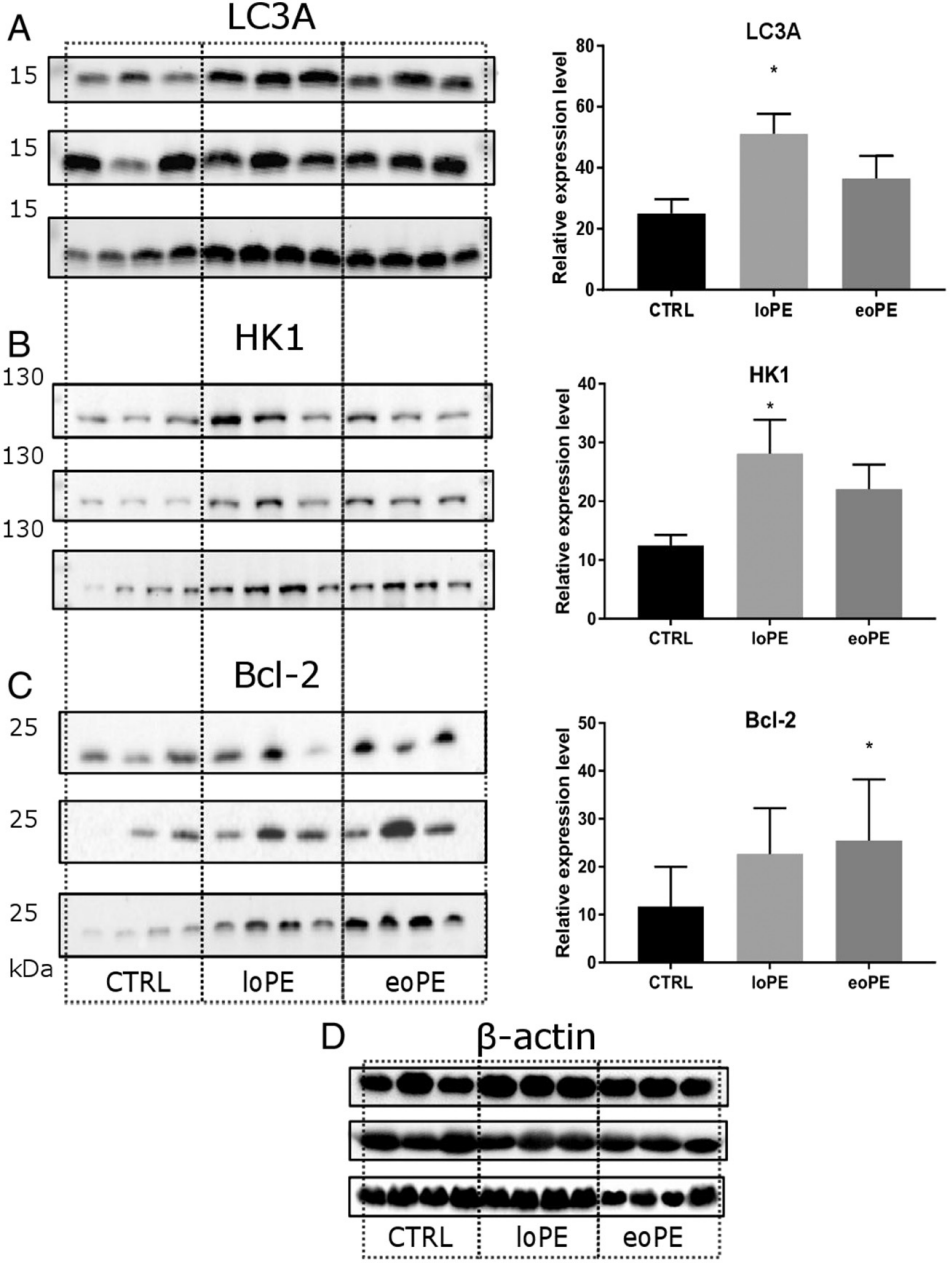
Autophagy in myometrium. Relative level of LC3A (A), HK1 (B) and Bcl-2 (C) proteins normalised on β-actin (left side – membrane staining, right side – protein level relative to β-actin). Protein expression was measured in myometrium from normal (CTRL) and preeclamptic pregnancies: late-onset PE – loPE, early-onset PE – eoPE (n = 10 for each group), by western blot analysis. β-actin was used as a loading control (D). *p < 0.05 versus control.

To verify whether autophagy is activated at PE we evaluated expression level of the autophagosomal membrane-bound form (LC3-II) of common autophagy marker LC3A/B, which combines both LC3 isoforms: A and B. We didn't observe significant difference (p = 0.081) in LC3A/B protein level among the groups (S2 Fig.). Measuring LC3A level separately (also LC3-II form), we found significant increase in the loPE group (p = 0.013), compared to the control (Fig. 4A). In contrast, western blot analysis did not reveal differences in the expression of proteins that are involved in the regulation of autophagy and its subtype – mitophagy. Production of Parkin, which takes part in the labeling of mitochondria for further mitophagy, PINK1 (PTEN-induced putative kinase 1; Parkin activation), p62 (adapter for autophagosome capture), Beclin1 (apoptosis and autophagy regulator), AMPK (key player in signaling pathway, possibly leading to autophagy) and BNIP3 (Bcl-2 family member, responsible for alternative mitophagy activation) was at the same level in all three groups tested (S3 Fig.). Interestingly, significant increase in hexokinase 1 (HK1) level was observed in the loPE group (p = 0.041), as shown in Fig. 4B. HK1 catalyses the first step of glycolysis and may be related to mitochondria labeling for further mitophagy. Furthermore, the level of Bcl-2 (Fig. 4C), which may regulate autophagy, was significantly higher in the eoPE group than the control (p = 0.017).

## 4 Discussion

Preeclampsia is a pregnancy-specific syndrome, which manifests as disturbances in circulatory, excretory, immune, visual and other organ system functions [25]. Despite the considerable number of studies into the placenta, other organs such as the adjacent uterus, may be involved in the progression of the disease. The myometrium is the muscle layer of the uterus, and is rich in vessels which pass blood from the mother to the fetus and vice versa. Oxidative stress markers in patients with PE are present not only in placental tissue, but also in maternal blood [10], [26], [27], [28].

In the present study, significant differences between preeclamptic myometrium and the control were observed in the levels of the proteins responsible for antioxidant defense, mitochondrial biogenesis and autophagy. We support the hypothesis about influence on maternal organism through extracellular messengers such as ROS, e.g. in the form of hydrogen peroxide [29], [30], lipid peroxides, also calcium [31], microparticles (exosomes and microvesicles) [32], [33] and cytokines circulating in the blood of the mother and fetus [10], [32], [34], [35]. The alterations in protein expression levels observed in preeclamptic myometrium, may be due to the activity of agents derived from the placenta or proinflammatory factors. Indeed, the myometrial biopsies were not extracted from the placental attachment site, supporting the hypothesis of a general influence to the myometrium and maternal circulation, rather than a local effect only in the placental bed area.

The problem of oxidative stress is in the focus of many works concerning PE. Therefore it was important for us to estimate the changes in content of antioxidant enzymes in preeclamptic myometrium in comparison to control group. In present study we investigated four antioxidant enzymes: SOD1, SOD2, catalase and GPx1. SOD1 is a component of an essential cytosolic system which catalyses the first step of the enzymatic conversion of superoxide radical to water, i.e., the reduction to hydrogen peroxide. Catalase is responsible for the further reduction of hydrogen peroxide to water and oxygen. The significant decrease of catalase and SOD1 levels observed in both PE groups, suggests a disruption in the activity of the “first line” of antioxidant defense, located mainly in the cytoplasm. This may be due to the influence of exogenous agents, which initially affect cytoplasmic enzymes. Kirbas et al. [36] observed significant decrease in the total antioxidant capacity in the blood of preeclamptic women. Similar associations concerning reduced SOD and catalase activities, and their mRNA levels have also been demonstrated in several other studies [26], [37], [38], [39], [40]. Although direct measurements of ROS in myometrial biopsies were not performed in our study due to its short lifespan, increases in PGC-1α and PGC-1β levels were observed, and may indicate oxidative stress [41]. St-Pierre et al. [42] demonstrated the up-regulation of PGC-1α and PGC-1β levels under oxidative stress conditions in a cell model. The imbalance between antioxidant enzymes content and ROS formation in maternal organism is a common and well-established feature of PE [7].

Reductions in oxidative metabolism and/or decline of antioxidant defense, lead to biomolecule and cellular component damage [43], [44]. Autophagy is a process whereby intracellular macromolecules and organelles are initially engulfed by autophagosomes, and then directed to lysosomes [45]. Activation of autophagy has been demonstrated in different human pathology states including cardiovascular diseases, ischemia and cancer [15], [46]. Elimination of accumulated oxidised molecules and organelles provides cell survival, by maintaining cellular homeostasis [15]. During autophagy, the PINK1-Parkin-mediated pathway promotes labeling of depolarised mitochondria by ubiquitin and the special adapter protein p62 [47]. Cytosolic protein LC3-I undergoes conversion to the LC3-II form, which conjugates to phosphatidylethanolamine on the autophagosomal membrane, and recognises a specific label on the surface of organelles (like p62) [48], [49]. After the fusion of autophagosome with lysosome, its internal contents (including LC3) are degraded by hydrolases. Several authors claim that the content of LC3 may reflect autophagic activity [49], [50], [51]. There are three cellular LC3 isoforms (LC3A, LC3B and LC3C) that are considered to have different localisation and probably – functions, despite their high similarity [52]. We found significant difference in LC3A level between normal and loPE myometrium, but not in LC3A/B level, a common autophagy marker. Nevertheless, LC3A has been shown to be involved in autophagy promotion, and may serve as an autophagy marker [51], [53], [54], [55], [56]. This may indicate an alternative autophagy pathway that involves LC3A as a major player in preeclamptic myometrium; further studies are required for confirmation. Our data is in agreement with work of Oh et al. [57] who observed a significant increase in LC3 levels associated with PE but in placentas. Another study showed elevated LC3 level in a primary culture of extravillous trophoblast cells under hypoxic condition [58].

Reduction in oxidative metabolism in PE due to the fluctuation of oxygen consumption, promotes enhanced ROS production and autophagy [57], [59], [60], [61]. Notably, an energy deficit may be partially balanced by activation of glycolysis. One of the key glycolytic enzymes, hexokinase, is responsible for hexoses entering the degradation pathway by its initial phosphorylation in a rate-limited manner, dependent on intracellular ATP concentration [62]. In our study, a significant increase in HK1 level was observed in the loPE group. Hexokinase is thought to bind to mitochondria through the VDAC1 docking site, and form metabolic compartments termed contact sites [63], [64]. Furthermore, HK1 may be ubiquitinated by Parkin during autophagy [47], [65], and is essential for Parkin relocalisation from the cytoplasm to depolarised mitochondria for labeling [66]. Indeed, VDAC1 (the level of which was also higher in the loPE group) may serve as a platform for ubiquitination and mitochondria selection in a Δψ-dependent manner during autophagy [47], [48], [67]. Thus, we suggest that increases in HK1 and VDAC1 levels in the loPE group could be linked to glucose metabolism, probably due to hypoxia or the activity of exogenous agents [68], and also to autophagy activation in loPE myometrium.

The essential mitochondrial protein TFAM is responsible for mtDNA transcription and replication, and its level was found to be elevated in the loPE group. This finding, combined with the significant increases in PGC proteins and VDAC1 levels in loPE myometrium, led us to expect the increase of mitochondrial content markers: mtDNA copy number and citrate synthase activity. However, such changes were not observed, probably due to the high rate of mitochondrial turnover promoted by autophagy or alternative regulation pathways. The lack of observable changes in mitophagy proteins (e.g., Parkin, PINK1, p62 and Beclin1) may be due to the initial high levels required for performance of its functions.

The absence of autophagy induction in the eoPE group may indicate the presence of an inhibition mechanism. Indeed, we observed a significant increase in Bcl-2 expression in the eoPE group. This anti-apoptotic protein may inhibit Beclin1 activity and consequently the autophagy process [69], [70]. In eoPE myometrium the significant increase in OPA1 mRNA level and the ratio of uncleaved to cleaved OPA1, indicates the prevailing of fusion to fission [71], [72], [73]. This result is in agreement with our previously study, which demonstrated an increase in OPA1 expression in placenta either in eoPE group [23].

The results suggest that different molecular mechanisms underlay early- and late-onset PE pathology. These may be sequential events of the same process resulting from an adaptive response to PE conditions. It is evident that there is a high regenerative capacity of placental stem cells during early gestation [74]. In eoPE myometrium we observed an increase in mitochondrial fusion with blocking of apoptosis and autophagy by Bcl-2, whilst in loPE, autophagy was activated. According to the model of mitochondria turnover described in the review of Twig et al. [75], mitochondrial fusion precedes autophagy. At late gestation age, the mechanism of oxidative stress compensation in loPE includes the induction of another autophagy pathway; this warrants further investigations.

## 5 Conclusions

The results of this study have revealed important alterations in the molecular machinery of preeclamptic myometrium with regard to antioxidant enzymes content, markers of mitochondrial biogenesis and autophagy.

## Conflict of interests

The authors declare that they have no conflict of interest.

## Authorship

Contribution: Conceived and designed the experiments: PAV, MYV, GTS. Performed the experiments: PAV, MAV, NVT, MVM. Analysed the data: PAV, MAV, NVT, MYV. Patient handling and human samples: NEK, ZSK. Wrote the manuscript: PAV, MYV.

## Transparency document

Transparency document. Image 1

## Appendix A Supplementary data

Supplementary material Image 2

## References

[1] Osungbade K.O., Ige O.K. (2011). Public health perspectives of preeclampsia in developing countries: implication for health system strengthening. J. Pregnancy.

[2] Lisonkova S., Sabr Y., Mayer C., Young C., Skoll A., Joseph K.S. (2014). Maternal morbidity associated with early-onset and late-onset preeclampsia. Obstet. Gynecol..

[3] Roberts J.M., Druzin M., August P.A., Gaiser R.R., Bakris G., Granger J.P. (2012). ACOG guidelines: hypertension in pregnancy. American College of Obstetricians and Gynecologists.

[4] Brosens I.A., Robertson W.B., Dixon H.G. (1972). The role of the spiral arteries in the pathogenesis of preeclampsia. Obstet. Gynecol. Annu..

[5] Meekins (1994). A study of placental bed spiral arteries and trophoblast invasion in normal and severe pre-eclamptic pregnancies. Br. J. Obstet. Gynaecol..

[6] Naicker T., Khedun S.M., Moodley J., Pijnenborg R. (2003). Quantitative analysis of trophoblast invasion in preeclampsia. Acta Obstet. Gynecol. Scand..

[7] Elliot M.G. (2016). Oxidative stress and the evolutionary origins of preeclampsia. J. Reprod. Immunol..

[8] D'Souza V., Rani A., Patil V., Pisal H., Randhir K., Mehendale S. (2016). Increased oxidative stress from early pregnancy in women who develop preeclampsia. Clin. Exp. Hypertens..

[9] Burton G.J., Yung H.W., Murray A.J. (2017). Mitochondrial – Endoplasmic reticulum interactions in the trophoblast: Stress and senescence. Placenta.

[10] Raijmakers M.T.M., Dechend R., Poston L. (2004). Oxidative stress and preeclampsia: rationale for antioxidant clinical trials. Hypertension.

[11] Hinrichsen Colin (1997). Organ Histology.

[12] Anton L., Merrill D.C., Neves L.A.A., Diz D.I., Corthorn J., Valdes G. (2009). The uterine placental bed renin-angiotensin system in normal and preeclamptic pregnancy. Endocrinology.

[13] Cooke C.L., Brockelsby J.C., Baker P.N., Davidge S.T. (2003). The receptor for advanced glycation end products (RAGE) is elevated in women with preeclampsia. Hypertens. Pregnancy.

[14] Faxén M., Nisell H., Kublickiene K.R. (2001). Altered mRNA expression of ecNOS and iNOS in myometrium and placenta from women with preeclampsia. Arch. Gynecol. Obstet..

[15] Navarro-Yepes J., Burns M., Anandhan A., Khalimonchuk O., del Razo L.M., Quintanilla-Vega B. (2014). Oxidative stress, redox signaling, and autophagy: cell death versus survival. Antioxid. Redox Signal..

[16] Wang Y., Walsh S. (2001). Increased superoxide generation is associated with decreased superoxide dismutase activity and mRNA expression in placental trophoblast cells in pre-eclampsia. Placenta.

[17] Manes C. (2001). Human placental NAD(P)H oxidase: solubilization and properties. Placenta.

[18] Zorov D.B., Juhaszova M., Sollott S.J. (2014). Mitochondrial reactive oxygen species (ROS) and ROS-induced ROS release. Physiol. Rev..

[19] Ye J., Coulouris G., Zaretskaya I., Cutcutache I., Rozen S., Madden T.L. (2012). Primer-BLAST: a tool to design target-specific primers for polymerase chain reaction. BMC Bioinf..

[20] Simon P. (2003). Q-Gene: processing quantitative real-time RT-PCR data. Bioinformatics.

[21] Venegas Victor, Halberg Michelle C., Wong L.-J.C. (2012). Mitochondrial disorders - biochemical and molecular analysis | Lee-Jun C. Wong | Springer. Mitochondrial Disord.

[22] Eigentler A., Draxl A., Wiethüchter A. (2015). Laboratory protocol: citrate synthase a mitochondrial marker enzyme. Mitochondrial Physiol. Netw. 04.

[23] Vishnyakova P.A., Volodina M.A., Tarasova N.V., Marey M.V., Tsvirkun D.V., Vavina O.V. (2016). Mitochondrial role in adaptive response to stress conditions in preeclampsia. Sci Rep.

[24] Shapiro S.S., Wilk M.B. (1965). An analysis of variance test for normality (complete samples). Biometrika.

[25] Sibai B., Dekker G., Kupferminc M. (2005). Pre-eclampsia. Lancet.

[26] D'Souza V., Rani A., Patil V., Pisal H., Randhir K., Mehendale S. (2016). Increased oxidative stress from early pregnancy in women who develop preeclampsia. Clin. Exp. Hypertens..

[27] Karacay O., Sepici-Dincel A., Karcaaltincaba D., Sahin D., Yalvaç S., Akyol M. (2010). A quantitative evaluation of total antioxidant status and oxidative stress markers in preeclampsia and gestational diabetic patients in 24–36 weeks of gestation. Diabetes Res. Clin. Pract..

[28] Kharb S. (2000). Low whole blood glutathione levels in pregnancies complicated by preeclampsia and diabetes. Clin. Chim. Acta..

[29] Pletjushkina O.Y., Fetisova E.K., Lyamzaev K.G., Ivanova O.Y., Domnina L.V., Vyssokikh M.Y. (2006). Hydrogen peroxide produced inside mitochondria takes part in cell-to-cell transmission of apoptotic signal. Biochemistry (Mosc).

[30] Pletjushkina O.Y., Fetisova E.K., Lyamzaev K.G., Ivanova O.Y., Domnina L.V., Vyssokikh M.Y. (2005). Long-distance apoptotic killing of cells is mediated by hydrogen peroxide in a mitochondrial ROS-dependent fashion. Cell Death Differ..

[31] Steinert J.R., Wyatt A.W., Jacob R., Mann G.E. (2009). Redox modulation of Ca^2 +^ signaling in human endothelial and smooth muscle cells in pre-eclampsia. Antioxid. Redox Signal..

[32] Pillay P., Maharaj N., Moodley J., Mackraj I. (2016). Placental exosomes and pre-eclampsia: Maternal circulating levels in normal pregnancies and, early and late onset pre-eclamptic pregnancies. Placenta.

[33] Gilani S.I., Weissgerber T.L., Garovic V.D., Jayachandran M. (2016). Preeclampsia and extracellular vesicles. Curr. Hypertens. Rep..

[34] Catarino C., Santos-Silva A., Belo L., Rocha-Pereira P., Rocha S., Patrício B. (2012). Inflammatory disturbances in preeclampsia: relationship between maternal and umbilical cord blood. J. Pregnancy.

[35] McCarthy C.M., Kenny L.C. (2016). Immunostimulatory role of mitochondrial DAMPs: alarming for pre-eclampsia?. Am. J. Reprod. Immunol..

[36] Kirbas A., Daglar K., Gencosmanoglu G., Yilmaz Z., Timur H., Inal Z. (2016). Total oxidative and anti-oxidative status, and ADAMTS-12 levels in placenta previa and early-onset severe preeclampsia. Pregnancy Hypertens..

[37] Bakacak M., Kılınç M., Serin S., Ercan Ö., Köstü B., Avcı F. (2015). Changes in copper, zinc, and malondialdehyde levels and superoxide dismutase activities in pre-eclamptic pregnancies. Med. Sci. Monit..

[38] de Lucca L., Rodrigues F., Jantsch L.B., Kober H., Neme W.S., Gallarreta F.M.P. (2016). Delta-aminolevulinate dehydratase activity and oxidative stress markers in preeclampsia. Biomed Pharmacother.

[39] Kaur G., Mishra S., Sehgal A., Prasad R. (2008). Alterations in lipid peroxidation and antioxidant status in pregnancy with preeclampsia. Mol. Cell. Biochem..

[40] Nakamura M., Sekizawa A., Purwosunu Y., Okazaki S., Farina A., Wibowo N. (2009). Cellular mRNA expressions of anti-oxidant factors in the blood of preeclamptic women. Prenat. Diagn..

[41] Lee R., Margaritis M., Channon K.M., Antoniades C. (2012). Evaluating oxidative stress in human cardiovascular disease: methodological aspects and considerations. Curr. Med. Chem..

[42] St-Pierre J., Drori S., Uldry M., Silvaggi J.M., Rhee J., Jäger S. (2006). Suppression of reactive oxygen species and neurodegeneration by the PGC-1 transcriptional coactivators. Cell.

[43] Uttara B., Singh A.V., Zamboni P., Mahajan R.T. (2009). Oxidative stress and neurodegenerative diseases: a review of upstream and downstream antioxidant therapeutic options. Curr. Neuropharmacol..

[44] Poljsak B., Šuput D., Milisav I., Milisav I. (2013). Achieving the balance between ROS and antioxidants: when to use the synthetic antioxidants. Oxidative Med. Cell. Longev..

[45] Kara A., Gedikli S., Sengul E., Gelen V., Ozkanlar S. (2016). Oxidative stress and autophagy. Free Radicals Dis.

[46] Lee J., Giordano S., Zhang J. (2012). Autophagy, mitochondria and oxidative stress: cross-talk and redox signalling. Biochem. J..

[47] Maejima Y., Chen Y., Isobe M., Gustafsson Å.B., Kitsis R.N., Sadoshima J. (2015). Recent progress in research on molecular mechanisms of autophagy in the heart. Am. J. Physiol. Heart Circ. Physiol..

[48] Geisler S., Holmström K.M., Skujat D., Fiesel F.C., Rothfuss O.C., Kahle P.J. (2010). PINK1/Parkin-mediated mitophagy is dependent on VDAC1 and p62/SQSTM1. Nat. Cell Biol..

[49] Tanida I., Ueno T., Kominami E. (2008). LC3 and autophagy BT - autophagosome and phagosome. Methods Mol. Biol..

[50] Kabeya Y., Mizushima N., Ueno T., Yamamoto A., Kirisako T., Noda T. (2000). LC3, a mammalian homologue of yeast Apg8p, is localized in autophagosome membranes after processing. EMBO J..

[51] Wan B. (2006). Molecular Cloning and Characterization of rat LC3A and LC3B - Two Novel Markers of Autophagosome.

[52] Koukourakis M.I., Kalamida D., Giatromanolaki A., Zois C.E., Sivridis E., Pouliliou S. (2015). Autophagosome proteins LC3A, LC3B and LC3C have distinct subcellular distribution kinetics and expression in cancer cell lines. PLoS One.

[53] Zois C.E., Giatromanolaki A., Sivridis E., Papaiakovou M., Kainulainen H., Koukourakis M.I. (2011). “Autophagic flux” in normal mouse tissues: Focus on endogenous LC3A processing. Autophagy.

[54] Sivridis E., Giatromanolaki A., Liberis V., Koukourakis M.I. (2011). Autophagy in endometrial carcinomas and prognostic relevance of “stone-like” structures (SLS): What is destined for the atypical endometrial hyperplasia?. Autophagy.

[55] Giatromanolaki A., Koukourakis M.I., Harris A.L., Polychronidis A., Gatter K.C., Sivridis E. (2010). Prognostic relevance of light chain 3 (LC3A) autophagy patterns in colorectal adenocarcinomas. J. Clin. Pathol..

[56] Choi J., Kim D.H., Jung W.H., Koo J.S. (2013). Metabolic interaction between cancer cells and stromal cells according to breast cancer molecular subtype. Breast Cancer Res..

[57] Oh S.-Y., Choi S.-J., Kim K.H., Cho E.Y., Kim J.-H., Roh C.-R. (2008). Autophagy-related proteins, LC3 and Beclin-1, in placentas from pregnancies complicated by preeclampsia. Reprod. Sci..

[58] Nakashima A., Yamanaka-Tatematsu M., Fujita N., Koizumi K., Shima T., Yoshida T. (2013). Impaired autophagy by soluble endoglin, under physiological hypoxia in early pregnant period, is involved in poor placentation in preeclampsia. Autophagy.

[59] Sanchez-Aranguren L.C., Prada C.E., Riato-Medina C.E., Lopez M. (2014). Endothelial dysfunction and preeclampsia: role of oxidative stress. Front. Physiol..

[60] Myatt L., Webster R.P. (2009). Vascular biology of preeclampsia. J. Thromb. Haemost..

[61] Gao L., Qi H.-B., Kamana K., Zhang X.-M., Zhang H., Baker P.N. (2015). Excessive autophagy induces the failure of trophoblast invasion and vasculature. J. Hypertens..

[62] Wilson J.E. (2003). Isozymes of mammalian hexokinase: structure, subcellular localization and metabolic function. J. Exp. Biol..

[63] Vyssokikh M.Y., Brdiczka D. (2003). The function of complexes between the outer mitochondrial membrane pore (VDAC) and the adenine nucleotide translocase in regulation of energy metabolism and apoptosis. Acta Biochim. Pol..

[64] Pastorino J.G., Hoek J.B. (2008). Regulation of hexokinase binding to VDAC. J. Bioenerg. Biomembr..

[65] Sarraf S.A., Raman M., Guarani-Pereira V., Sowa M.E., Huttlin E.L., Gygi S.P. (2013). Landscape of the PARKIN-dependent ubiquitylome in response to mitochondrial depolarization. Nature.

[66] McCoy M.K., Kaganovich A., Rudenko I.N., Ding J., Cookson M.R. (2014). Hexokinase activity is required for recruitment of parkin to depolarized mitochondria. Hum. Mol. Genet..

[67] Gatliff J., East D., Crosby J., Abeti R., Harvey R., Craigen W. (2014). TSPO interacts with VDAC1 and triggers a ROS-mediated inhibition of mitochondrial quality control. Autophagy.

[68] Waskova-Arnostova P., Kasparova D., Elsnicova B., Novotny J., Neckar J., Kolar F. (2014). Chronic hypoxia enhances expression and activity of mitochondrial creatine kinase and hexokinase in the rat ventricular myocardium. Cell. Physiol. Biochem..

[69] Marquez R.T., Xu L. (2012). Bcl-2:Beclin 1 complex: multiple, mechanisms regulating autophagy/apoptosis toggle switch. Am. J. Cancer Res..

[70] Pattingre S., Tassa A., Qu X., Garuti R., Liang X.H., Mizushima N. (2005). Bcl-2 antiapoptotic proteins inhibit Beclin 1-dependent autophagy. Cell.

[71] El-Sikhry H.E., Alsaleh N., Dakarapu R., Falck J.R., Seubert J.M. (2016). Novel roles of epoxyeicosanoids in regulating cardiac mitochondria. PLoS One.

[72] Song Z., Chen H., Fiket M., Alexander C., Chan D.C. (2007). OPA1 processing controls mitochondrial fusion and is regulated by mRNA splicing, membrane potential, and Yme1L. J. Cell Biol..

[73] Alavi M.V., Fuhrmann N. (2013). Dominant optic atrophy, OPA1, and mitochondrial quality control: understanding mitochondrial network dynamics. Mol. Neurodegener..

[74] Weber M., Göhner C., San Martin S., Vattai A., Hutter S., Parraga M. (2016). Unique trophoblast stem cell- and pluripotency marker staining patterns depending on gestational age and placenta-associated pregnancy complications. Cell Adhes. Migr..

[75] Twig G., Hyde B., Shirihai O.S. (2008). Mitochondrial fusion, fission and autophagy as a quality control axis: the bioenergetic view. Biochim. Biophys. Acta.

