# Supplementary material for: Alterations in antioxidant system, mitochondrial biogenesis and autophagy in preeclamptic myometrium
Source: BBA Clin. 2017 Jul 3;8:35–42. doi: 10.1016/j.bbacli.2017.06.002 (PMC5512187; doi:10.1016/j.bbacli.2017.06.002)
Supplement: Supplementary file 2 — Supplementary material [file mmc2.pdf]

SUPPLEMENTARY MATERIAL

Alterations in antioxidant system, mitochondrial biogenesis and autophagy in preeclamptic myometrium.

Polina A. Vishnyakova, Maria A. Volodina, Nadezhda V. Tarasova, Maria V. Marey, Natalya E. Kan, Zulfiya S. Khodzhaeva, Mikhail Yu. Vysokikh and Gennady T. Sukhikh

Supplementary Table S1. Primer sequences.

Gene	Primer sequence: sense and antisense	Product size (bp)
ACTB	5` GAGCGGGAAATCGTGCGTGACATT 3`<br>5` GATGGAGTTGAAGGTAGTTTCGTG 3`	234
OPA1	5` GTACCGTTAGCCCTGAGACC 3`<br>5` AGCCATGCCTGATGTCACAG 3`	110
NRF1	5` GTGGGGGACAGATCGTCTTG 3`<br>5` ATCTGGACCAGGCCATTAGC 3`	88
NFE2L2	5` AGTGTGGCATCACCAGAACA 3`<br>5` TGTTTGACACTTCCAGGGGC 3`	113
**mtDNA (D-loop)**	5` ATCCCGCACAAGAGTGCTAC 3`<br>5` GGGGAACGTGTGGGCTATTT 3`	124
B2M	5` AAAAGATGAGTATGCCTGCCG 3`<br>5` TGCAGAGTGTTATATCAGATGGAT 3`	150

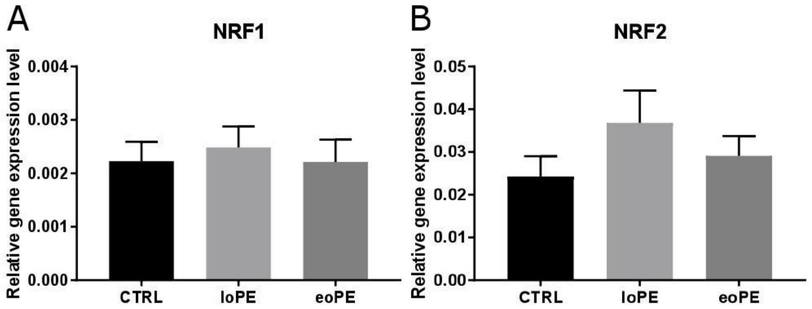

S1 Figure. Expression level of NRF1 and NRF2 genes in myometrium from control (CTRL) and preeclamptic pregnancies: late-onset PE – loPE, early-onset PE – eoPE, according to the results of PCR analysis (n=10 for each group).

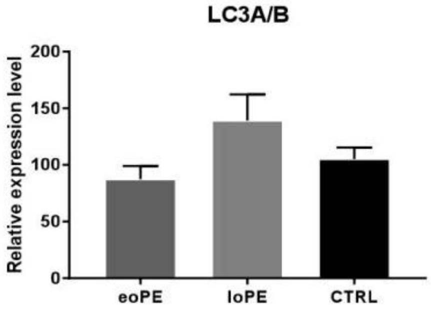

S2 Figure. Expression level of LC3A/B proteins in myometrium from normal (CTRL) and preeclamptic pregnancies: late-onset PE – loPE, early-onset PE – eoPE. Relative expression level of LC3A/B is shown according to the results of western blot analysis (n=10 for each group). Staining was performed with the following anti-LC3A/B antibodies: ab128025 – Abcam, USA; 12741 – Cell signaling, USA.

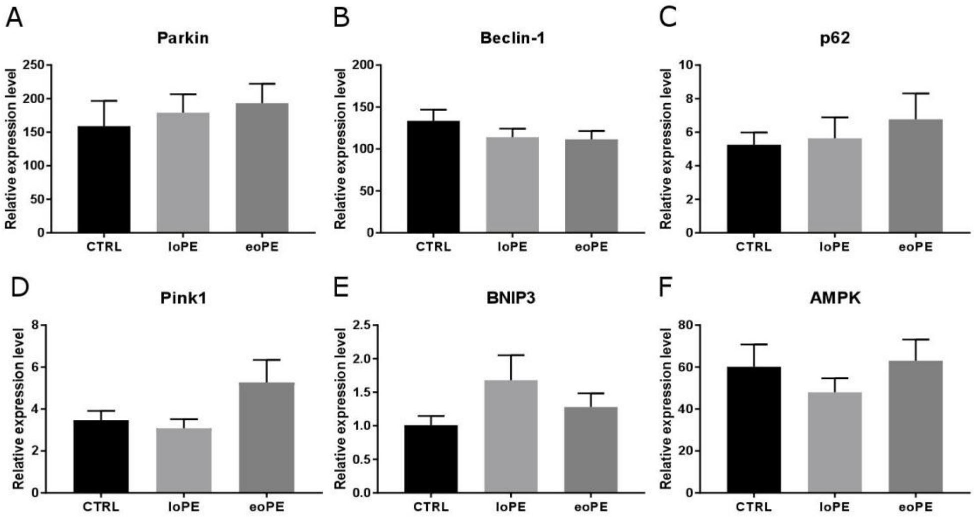

S3 Figure. Expression level different proteins, involving in autophagy release, in myometrium from normal (CTRL) and preeclamptic pregnancies: late-onset PE – loPE, early-onset PE – eoPE. Relative expression level of Parkin (A), Beclin-1 (B), p62 (C), Pink1 (D), BNIP3 (E) and AMPK (F) is shown according to the results of western blot analysis (n=10 for each group). Staining was performed with the following antibodies: anti-Parkin – ab15954; anti-Pink1 – ab23707; anti-BNIP3 – ab109362; anti-AMPK – ab80039; Abcam, USA; anti-Beclin-1 – 3738S; p62 – 8025S; Cell signaling, USA.
